# Fluorescence enhancement of gold nanoclusters *via* Zn doping for biomedical applications†

**DOI:** 10.1039/c7ra13072d

**Published:** 2018-02-15

**Authors:** Yanqing Qiao, Ying Liu, Haixia Liu, Yonghui Li, Wei Long, Junying Wang, Xiaoyu Mu, Jing Chen, Haile Liu, Xueting Bai, Lingfang Liu, Yuan-Ming Sun, Qiang Liu, Meili Guo, Xiao-Dong Zhang

**Affiliations:** Department of Physics, Tianjin Key Laboratory of Low Dimensional Materials Physics and Preparing Technology, School of Sciences, Tianjin University Tianjin 300350 China xiaodongzhang@tju.edu.cn; Tianjin Key Laboratory of Molecular Nuclear Medicine, Institute of Radiation Medicine, Chinese Academy of Medical Sciences, Peking Union Medical College No. 238, Baidi Road Tianjin 300192 China; Tianjin Collaborative Innovation Center of Chemical Science and Engineering Tianjin 300072 China; Department of Physics, School of Science, Tianjin Chengjian University Tianjin 300384 China meiliguo314@163.com

## Abstract

Gold nanoclusters (NCs) have been widely used in bioimaging and cancer therapy due to their unique electronic structures and tunable luminescence. However, their weak fluorescence prevents potential biomedical application, and thus it is necessary to develop an effective route to enhance the fluorescence of gold NCs. In this work, we report the fluorescence enhancement of ultrasmall GSH-protected Au NCs by Zn atom doping. The fluorescence signal of Zn-doped Au NCs shows approximately 5-fold enhancement compared to pure Au NCs. Density functional theory (DFT) calculation shows that Zn doping can enhance the electronic states of the highest occupied molecular orbital (HOMO), leading to enhancement of visible optical transitions. *In vitro* experiments show that AuZn alloy NCs can enhance the cancer radiotherapy *via* producing reactive oxygen species (ROS) and don't cause significant cytotoxicity. *In vivo* imaging indicates AuZn alloy NCs have significant passive targeting capability with high tumor uptake. Moreover, nearly 80% of GSH-protected AuZn alloy NCs can be rapidly eliminated *via* urine excretion.

## Abstract 2

Fluorescence enhancement of gold nanoclusters *via* Zn doping.

## Graphical abstract

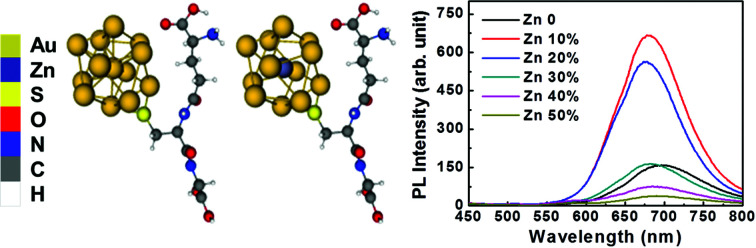

## Introduction

Gold nanoclusters (NCs), have been widely used in biological imaging, cancer therapy, energy and environmental science,^1–12^ owing to their numerous peculiar features such as unique size^13^ and morphology-dependent optical, catalytic, and electronic properties.^14–18^ Ultrasmall metal NCs, about 1–3 nm between single atoms and large nanocrystals,^19–21^ show quantum electronic properties and tunable fluorescence, and are expected to be potential candidates for novel fluorescent probes. One of the exciting properties of gold NCs is their fluorescence emission, which is related to their molecular-like properties, including intrinsic structure, composition, core size, and environment,^14,22–27^ and has attracted wide attention in tumor and related disease imaging.^28–30^ Besides, compared with traditional nanoparticles, the gold NCs can be cleaned by renal clearance and thus of minimized toxicity.^31–33^

As concerns the basic essence of luminescence, some researchers conjecture ligand-to-metal charge transfer (LMCT) and/or ligand-to-metal–metal charge transfer (LMMCT).^34^ Alloying or doping of the metal core of NCs with another metal element provides an effective method for improving the fluorescence.^8,18,33,35–41^ Recently, alloy nanoparticles and NCs receive significant attention and their synthesis and characterization also have achieved great progress, owing to their synergistic effects and enhanced properties in fluorescence compared to their mono-metallic NCs.^15,40–43^ For example, the fluorescence quantum yield (QY) of Au_25_ NCs doped with 13 silver atoms indicated nearly 200-fold enhancement.^8^ A 26-fold fluorescence enhancement by gold doping of silver NCs was observed.^38^ Bi-metallic Pt_1_Ag_28_(SR)_18_(PPh_3_)_4_ clusters were obtained and achieved nearly 50-fold enhancement of fluorescence.^18^ Therefore, we expect that doping with the other element can improve the fluorescence of Au NCs and reveal their fluorescence mechanism.

In this work, we report a facile one-pot synthesis strategy to prepare a water-soluble Zn doped Au NCs with an enhanced fluorescence. In addition, glutathione (GSH) is used as ligand molecule, which not only endows the clusters better biocompatibility but also enhances the anti-oxidation effects. The optical properties, stability, structure and composition of the NCs were investigated in detail through UV-vis spectrophotometer, fluorescence spectra, transmission electron microscopy, X-ray photoelectron spectroscopy and mass spectrum. Moreover, TDDFT simulation of model cluster systems was performed. Finally, a series of *in vitro* and *in vivo* biological experiments were conducted, indicating that it has great potential applications in radiation therapy.

## Experimental section

### Synthesis of clusters

NCs were synthesized in ultrapure water (18.2 MΩ) at room temperature under air atmosphere. Aliquots of reagents were added (in the following order) to a round bottomed flask containing a stir bar: 8.6 mL ice water, HAuCl_4_ (20.0 mM), Zn(NO_3_)_2_ (20.0 mM), GSH (750 μL, 20.0 mM), CsOH (25 μL, 50% w/w) and NaBH_4_ (900 μL, 20.0 mM). The total concentration of metal cations was held constantly while the molar ratio of gold and zinc was varied. The total volume of HAuCl_4_ and Zn(NO_3_)_2_ is 376 μL. After thoroughly mixing the gold and zinc solution by stirring for ∼10 s, GSH (750 μL, 20.0 mM) was quickly added to the solution and briefly mixed. With adding of CsOH, the solution color changed from yellow to clear. The metal and capping ligand precursor solution was immediately reduced by a rapid injection of NaBH_4_ (900 μL, 20.0 mM) solution while vigorously stirring (1150 rpm) for 5 min. The capped vials were stood for at least 1 hour. The cluster solution was concentrated using 15 mL ultrafiltration tubes with molar weight cut-off (MWCO) 10k and 30k, then the clusters with molar weight 10k–30k were collected and washed 3 times by ultrapure water in 3k ultrafiltration tubes.

### Materials characterization

UV-vis absorption spectra were recorded from a UV-3600 spectrophotometer (Jindao, Japan). Fluorescence spectra were acquired by Fluorolog3 (HORIBA, America). Transmission electron microscopy (TEM) images were conducted with JEM-2100F microscope (JEOL, Japan) at 200 kV. MALDI-TOF mass spectrum was acquired on Bruker Autoflex III TOF/TOF200. FT-IR spectra were determined with AVATAR 360. Fluorescence lifetime was measured by FLS920P fluorescence spectrometer. Inductively coupled plasma mass spectrometry (ICP-MS) was acquired by Agilent 7700X (Agilent, America). The valence states of elements were characterized by XPS. Zeta potential and hydrodynamic diameters were determined by NanoZS Zetasizer (Malvern).

### Fluorescence quantum yield of NCs

Fluorescence quantum yield (QY) of the NCs in water was measured using Cy5 as the reference (quantum yield 20%). The excitation wavelength of NCs was 270 nm and Cy5 was 650 nm. Then absorption spectra and fluorescence spectra of the dye solution and the NCs sample were measured respectively. The quantum yield of the sample was calculated by the equation as follows:
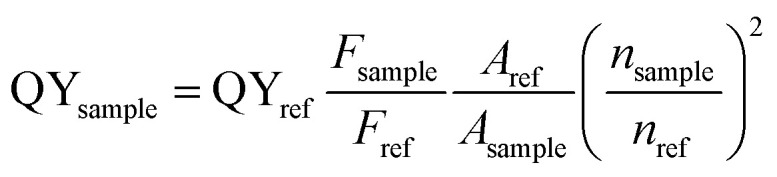
QY_sample_, QY_ref_ – the fluorescence quantum yield of test sample and the reference standard substance; *F*_sample_, *F*_ref_ – is the integrated fluorescence intensity of the test sample and the reference substance; *A*_ref_, *A*_sample_ – the fluorescence intensity of the test sample and the reference substance in the excitation; *n*_sample_, *n*_ref_ – is the refraction index of the test sample and the reference standard substance.

### *In vitro* cellular toxicity and viability

#### Cell toxicity

Chinese hamster ovary (CHO) cells were conducted in cell viability experiments. We prepared two 96-well plates with about 4000 cells per well (dissolved in DMEM with 10% FBS) and kept them into the atmosphere of 5% CO_2_ at 37 °C for 24 h. Therewith, 100 μL DMEM (10% FBS) containing AuZn alloy NCs (at different concentrations of 0, 3.75, 7.5, 15, 30, 60, 120, 240, and 480 μg mL^−1^) were employed to treat cells. After incubation for 24 and 48 h, cell viability was analyzed by MTT method. 10 μL of MTT reagent at 5 mg mL^−1^ was added into per well and further incubated for 4 h. Then media was discarded and followed by adding 150 μL of DMSO into each well to dissolve formazan crystals, and the optical absorption value was recorded at 490 nm by a microplate reader.

#### Radiation-related viability

CHO cells were planted into three 96-well plates with about 4 × 10^3^ cells per well and incubated for 24 h at 37 °C with 5% CO_2_. Next, the cells were treated with Zn-doped Au NCs or fresh culture medium, exposed to 0, 2 and 4 Gy radiation respectively, and further incubated for 24 h. The cell viability was estimated by MTT assay.

### Intracellular reactive oxygen species (ROS) evaluation

CHO cells were dispensed in three six-well plates with 1 × 10^5^ cells per well, treated with suitable DMEM (with 10% FBS) culture medium for 24 h. We chose one plate as control group, and the other two plates were treated with or without AuZn alloy NCs before 4 Gy radiation. After further 24 h incubation, media were replaced by 1 mL of 5 μM 2,7-dichlorofluorescein diacetate (DCFH-DA). Then, the cells were incubated for 20 min and washed three times with PBS. The fluorescence intensity was recorded at 488 nm excitation by a BD flow cytometer, and the cells were imaged by fluorescent microscope.

### *In vivo* biodistribution of AuZn alloy NCs

This study was performed in strict accordance with the Chinese Academy of Medical Sciences (CAMS) guidelines for the care and use of laboratory animals and was approved by Animal Care and Use Committee (IACUC), and handled under the Institute of Radiation Medicine, Chinese Academy of Medical Sciences (Tianjin, China).

Male BALB/c-nu mice were subcutaneously injected 2 × 10^6^ Hela cells (in 50 μL of PBS) into the right shoulders to generate tumour models. The content of NCs accumulated within tumours and other organs *in vivo* were analyzed by their fluorescence intensity. At 1, 2, 8 and 48 h after intravenous injection of AuZn alloy NCs, mice were anesthetized and imaged fluorescent signals from tumors and other organs of interest (heart, spleen, liver, lung, kidney, testis, and bladder). *In vivo* and *ex vivo* fluorescence imaging experiments were recorded by a Maestro *in vivo* imaging system (excitation: 435–480 nm, emission: 500–720 nm).

### Theoretical simulation of Zn-doped Au NCs

The quantum effect by the Zn doping is investigated by the TDDFT simulations theoretically. The simulation is done on XSEDE using Gaussian09.^44^ Unfortunately, the crystallization of GSH-protected NCs cannot be done successfully. So we do not have the coordinate and geometry of the NCs. To model the NCs, we adopt a well-known Au cluster core as an icosahedron formed by 13 gold atoms according to the literature^45^ and attached a single GSH ligand to the core. Similarly, a second model with zinc replaced core is also calculated. Thus, the zinc dopants effect can be studied by benchmarking the 2 models. Before any optical properties are simulated, we optimized the geometry of the ligands in the models with respect to a fixed gold core using M06-2x as the exchange–correlation functional.

### Statistical analysis

Data presented in this research is given as average ± SD. Independent Student's *t* test was used for statistical analysis.

## Results and discussion

**Fig. 1:**
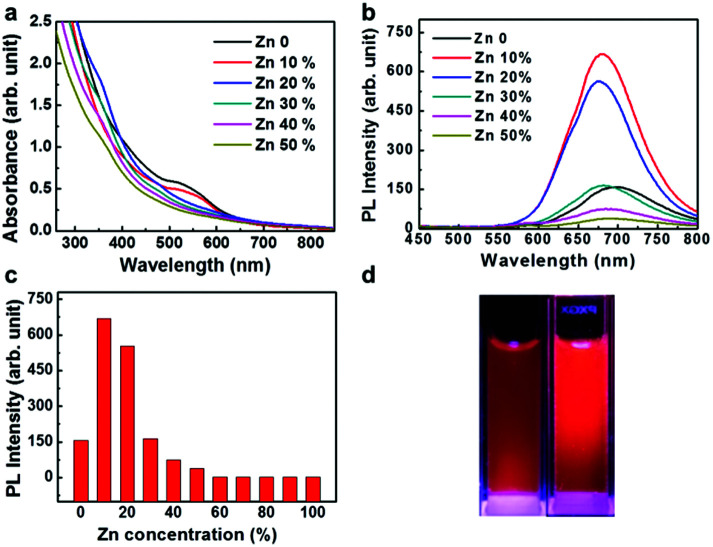
Optical characterization of Zn-doped Au NCs. (a) UV-vis absorption spectra of Au NCs with different Zn doping contents. (b and c) Fluorescence spectra (*λ*_ex_ = 270 nm) of Zn-doped Au NCs, synthesized with different molar ratios of Zn-to-Au precursor. (d) Images of Au NCs and AuZn alloy NCs under UV light.

UV-vis absorption spectra of Zn-doped Au NCs at different percentages are shown in Fig. 1a. For the pure Au NCs (black line) and 10% Zn-doped NCs, only one significant peak appears at 525 nm. However, the absorption intensity of gold clusters decreases after Zn doping compared with pure Au NCs. With increasing Zn doping concentration, the absorption spectra changes obviously. The absorption peak at 525 nm disappears and the new absorption peak at 350 nm appears. The Zn atoms are successfully doped into the clusters according to the variation in the absorption spectra. In order to confirm the optimized doping concentration for the fluorescence NCs, the corresponding fluorescence spectra of as-prepared NCs at different doping concentration have been tested as shown in Fig. 1b and c. Compared to the pure Au NCs, the emission bands of Zn-doped NCs appear obvious blue shift from 700 nm to 675 nm under the excitation wavelength at 270 nm. Fig. 1b displays the fluorescence peak of 10% Zn-doped NCs is highest among all the clusters. Fluorescence intensity of 10% Zn-doped NCs shows significant enhancement in Fig. 1c, approximately 5-fold higher than that of the pure Au NCs. As a result, the corresponding fluorescence QY is increased from 0.59 to 1.54% (about 2.6 times), which would be beneficial to biological applications. Based on the above results, 10% Zn-doping is the optimized doping concentration for fluorescence enhancement. So in the following, the sample is mainly characterized and tested. And it is unified labeled as AuZn alloy NCs as a matter of convenience. Meanwhile, images of pure Au NCs (left) and AuZn alloy NCs (right) under UV light are shown in Fig. 1d. In addition, the absorbance and fluorescence of GSH-protected AuZn alloy NCs displays superior stability in biological media such as water and phosphate buffer saline (PBS), and satisfactory photostability without significant decay as demonstrated in Fig. S1.† The zeta potential and hydrodynamic diameters of clusters in deionized ultrafiltered (DIUF) water or fetal bovine serum (FBS) were determined by dynamic light scattering (DLS) with a NanoZS Zetasizer (Malvern). The zeta potentials of the freshly prepared Zn doped Au NCs in H_2_O and FBS are −18.6 and −9.4 mV, respectively, as shown in Table S1.† After 48 h, The zeta potentials displays a little decrease in H_2_O but nearly no change in FBS, indicating the relative colloidal stability of the NCs. In addition, the stability of the Zn-doped Au NCs has been also demonstrated by the results from the hydrodynamic size in H_2_O and FBS (Fig. S2†).

**Fig. 2:**
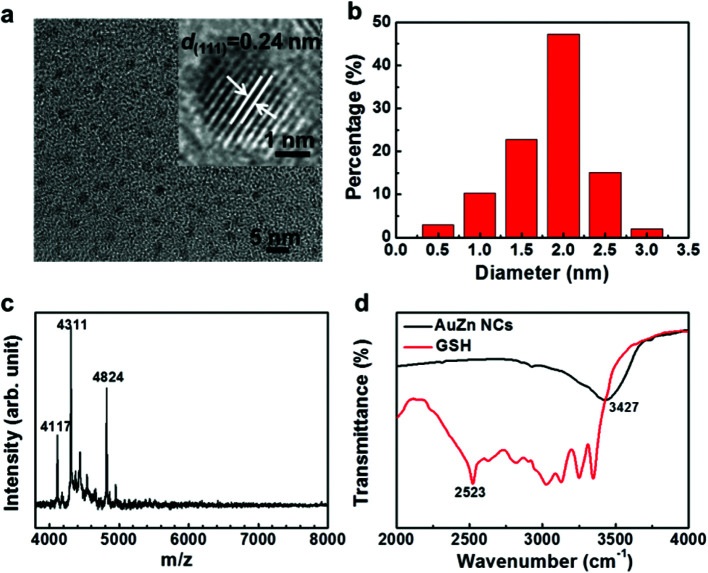
Structural characterization of GSH-protected AuZn alloy NCs. (a) HRTEM image; (b) size distribution obtained from HRTEM image with an average size of 1.84 nm. (c) MALDI TOF mass spectrum (positive mode) of GSH-protected AuZn alloy NCs. (d) FT-IR spectra obtained from pure GSH (black line) and GSH-protected AuZn alloy NCs (red line).

The size distribution of fluorescence alloy NCs was characterized by resolution transmission electron microscopy (TEM). TEM image of the AuZn alloy NCs is presented in Fig. 2a. High-resolution TEM shows a lattice distance of 0.24 nm, corresponding to the lattice plane (111) of Au. Apparently, no large NCs could be observed and their average diameter is about 1.84 nm obtained from statistical analysis in Fig. 2b. In order to determine the actual doping ratio of Zn, the alloy NCs was analyzed by XPS and ICP-MS. In the XPS spectrum of Au 4f (Fig. S3†), two peaks located at 83.6 and 87.3 eV, are corresponding to Au 4f_7/2_ and Au 4f_5/2_. XPS spectrum of Zu also has two peaks locked at 1022.4 and 1045.2 eV are ascribed to Zn 2p_3/2_ and Zn 2p_1/2_. XPS results also suggest Zn have been successfully doped into NCs and the molar ratio of Zn/Au is about 3.5%, which is close to the result of ICP-MS (3%). Further, the molecule weight of GSH-protected AuZn alloy NCs is detected by MALDI-TOF mass spectrum. The GSH-protected AuZn NCs (Fig. 2c) display several sets of intense peaks in the range of 4–5 kDa, which could be assigned to the complex of Au_21_Zn(SG)_17_, Au_18_Zn_2_(SG)_16_ and Au_17_Zn(SG)_14_, and it is similar to previous work.^46^ The GSH ligand shows strong affinity to the Au atom and is easy to form the metal complex, which leads to difficulty to determine the exact crystal structure.^4^Fig. 2d shows the FT-IR spectra of the free GSH (red line) and the fluorescent NCs samples (black line). Compared these two lines, it confirms that the characteristic band at 2523 cm^−1^ for *ν*(S–H) of GSH is disappeared, which is ascribed to the formation of S–Au or S–Zn bonds instead of S–H bonds.

**Fig. 3:**
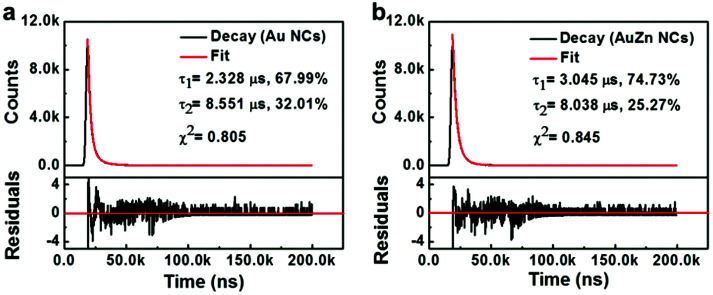
Fluorescence lifetime of (a) Au NCs and (b) AuZn alloy NCs.

The fluorescence emission of metal clusters is regarded as LMMCT relaxation occurring at a triplet metal-centered state *via* the comparatively long lifetime (microsecond-scale).^47^ The fluorescence decay spectra of the pure Au NCs and AuZn alloy NCs are measured as shown in Fig. 3. Both of two NCs can be fitted with a biexponential curve, which consists of two components with life times of *τ*_1_ = 2.328 μs (67.99%) and *τ*_2_ = 8.551 μs (32.01%) for Au NCs, *τ*_1_ = 3.045 μs (74.73%) and *τ*_2_ = 8.0381 μs (25.27%) for AuZn alloy NCs, respectively. For Zn doped Au NCs, the fluorescence decay is almost identical to pure Au NCs. The long fluorescence lifetime of the NCs may be ascribed to aggregation-induced emission (AIE).^4^ Long fluorescence lifetime and large Stokes shift (>200 nm) suggest the potential advantages on biological imaging.

**Fig. 4:**
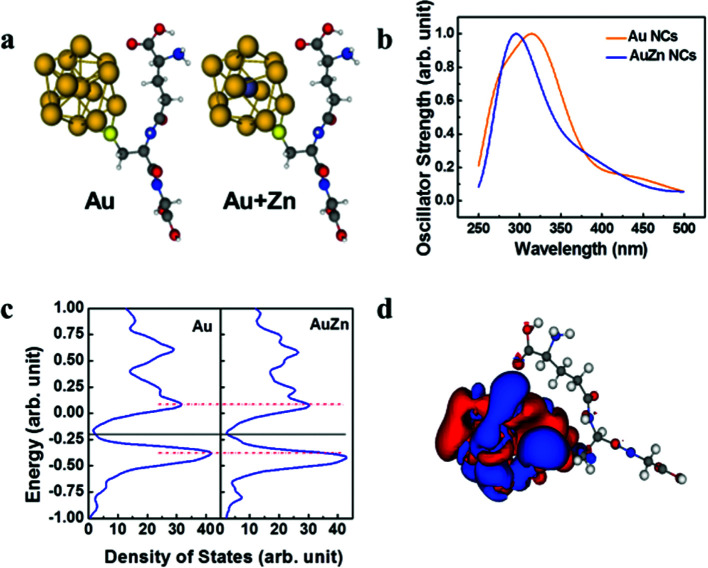
TDDFT simulation of model cluster systems of a metal core and one ligand. (a) Optimized geometry of the model system: gold core +1 ligand (left) and gold core with zinc central replacement +1 ligand (right). The gold 13 core is taken from literature. (b) Simulated absorption spectra of Au NCs and AuZn NCs. (c) Simulated density of states which is created from the Kohn–Sham energy with broadening factor 0.05 eV. (d) Density responds for Au + Zn model excited at 427 nm which corresponds to the excitation peak.

As shown in Fig. 4a, models with Au 13 core and Au 12 + 1 Zn core with a single GSH ligand attached are used to approach the optical properties of the clusters. The model systems are simulated by TDDFT with M06-2x as the exchange–correlation potential. The absorption spectra are shown in Fig. 4b. The theoretical model successfully reproduces the low energy behavior of the experimental absorption spectrum of Fig. 1a. This clearly indicates the effect of Zn doping, *i.e.* blue shift of the absorption spectrum. We then simulated the density of state using the Kohn–Sham energies with broadening factor 0.05 (Fig. 4c). The band gap of Zn doped model is slightly smaller than the undoped model. In addition, the undoped model shows an extra absorption peak near 427 nm compared to the Zn doped model. The iso-surface of the density responds for the undoped model at 427 nm is also provided (Fig. 4d). The electron probability density fluctuation is concentrated to the metal core rather than spread over the entire model which is similar to the other model.

**Fig. 5:**
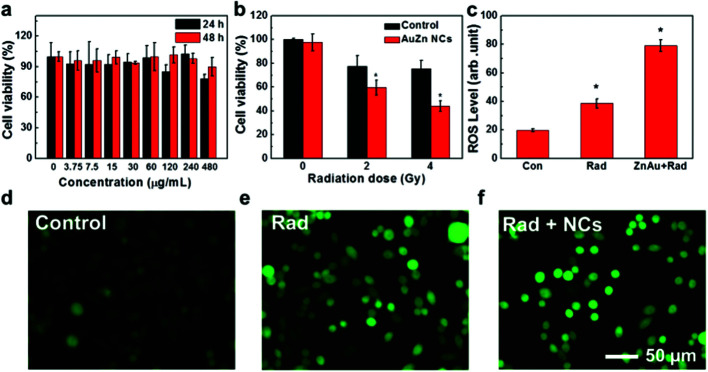
Cytotoxicity and radiosensitizing effect of AuZn alloy NCs *in vitro*. (a) Cell viabilities of AuZn alloy NCs at doses from 0 to 480 μg mL^−1^ after 24 and 48 h. (b) Cell viabilities without and with AuZn alloy NCs under radiation doses of 0, 2, and 4 Gy. (c) Quantitative analysis of intracellular ROS levels. (d–f) Fluorescence images of intracellular ROS levels.

The toxicity of clusters was assessed by testing the viability of Chinese hamster ovary (CHO) cells upon exposure to AuZn alloy NCs using MTT assay. GSH-protected AuZn alloy NCs do not cause obvious decrease of cell viability at the concentration up to 480 μg mL^−1^ after incubation of 24 and 48 h as shown in Fig. 5a, indicating their low cytotoxicity. GSH is widely applied as a ligand in nano-carrier system or protectors in biological applications, and it is endowed with low toxicity and great biocompatibility.^48^ We also detected the radiation dose-dependent cell viabilities and the radiosensitization effect of GSH-protected AuZn alloy NCs *in vitro* using MTT assays (Fig. 5b). The viabilities of CHO cells without AuZn alloy NCs sharply decrease with increasing radiation doses. After incubation of GSH-protected AuZn alloy NCs, the viabilities of irradiated cells decrease more sharply than only-irradiated cells without AuZn alloy NCs treatment at both radiation doses, suggesting its significant inhibiting effect on cell proliferation. Furthermore, in order to directly monitor radiation-induced intracellular ROS levels with and without treatment of GSH-protected AuZn alloy NCs, cellular imaging is performed by fluorescence microscopy (Fig. 5d–f). Healthy cells show weak fluorescence, suggesting low ROS level, while the only-irradiated cells exhibit strong fluorescence corresponding to high ROS level due to radiation damages. The fluorescence signal of irradiated cells with incubation of GSH-protected AuZn alloy NCs becomes much brighter than only irradiated cells, suggesting the ability of ROS proliferation.^49,50^Fig. 5c gives quantitative investigation of the ROS levels by flow cytometer, demonstrating that GSH-protected AuZn alloy NCs could achieve the cancer radiation-sensitizing therapy.

**Fig. 6:**
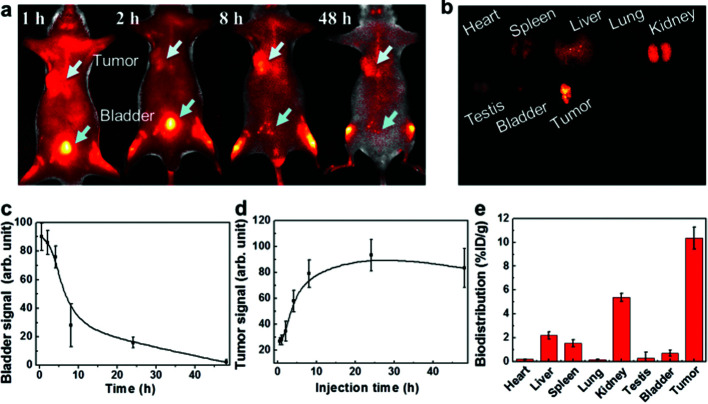
*In vivo* imaging, tumor uptake, renal clearance and biodistribution. (a) Fluorescence images *in vivo*. (b) *Ex vivo* fluorescence imaging of heart, liver, spleen, lung, kidney, testes, bladder and tumor. Fluorescence signal of the AuZn alloy NCs in tumor (c) and bladder (d) at different time points p.i. (e) Biodistribution of the mice treated with AuZn alloy NCs at after 48 h p.i.

Targeting capability and rapid excretion are important for minimizing damage to normal tissues during the cancer radiation treatment. The tumor uptake and bladder excretion of GSH-protected AuZn alloy NCs were investigated by analyzing the fluorescence signal of GSH-protected AuZn alloy NCs in the tumor tissue at different time points post injection (p.i.). As presented in Fig. 6a, the fluorescence signal in the tumor increases significantly from 1 to 10 h p.i., and gradually reaches a maximum at 24 h p.i. The stronger fluorescence could retain up to 48 h p.i. according to Fig. 6d. Simultaneously, the fluorescence signal in the bladder rapidly decreases from 1 to 10 h p.i. No fluorescence signal can be seen at 48 h p.i. (Fig. 6c), indicating rapid renal clearance. The ultrahigh tumor uptake and bladder excretion of GSH-protected AuZn alloy NCs could be attributed to their distinctive structure and ultrasmall size.^30^ In addition, to further understand the *in vivo* behavior of GSH-protected AuZn alloy NCs, we also investigated their biodistribution at 48 h p.i. (Fig. 6b and e). The uptake of GSH-protected AuZn alloy NCs in tumor tissue is much higher than that in all other key organs including kidney and liver. The high targeting specificity of GSH-protected AuZn alloy NCs to tumor implies that it could effectively enhance tumor radiotherapy and evade possible damages to normal tissues. Fast clearance and minimized toxicity are very important for all biomedical applications.^30,51^*In vivo* experiment demonstrates that the synthesized GSH-protected AuZn alloy NCs with ultrasmall size can be quickly cleared by kidneys.

Fluorescence is a most fascinating feature of NCs owing to great potential in biomedical applications. The present synthetic route of AuZn alloy NCs shows remarkable enhancement in fluorescence compared to pure Au NCs, which is useful for cellular imaging.^52,53^ The enhancement of fluorescence is ascribed to the changes of geometry and electronic structures by Zn doping.^14,54–57^ To enhance fluorescence by metal doping, many researchers focus on the Ag doping. Here, we find that the Zn doping can also enhance the fluorescence, so it is interesting to try other systems in the future. Besides doping of metal ions, nonmetal element doping is promising as a strategy of fluorescence enhancement as well. Ultrasmall size and rapid renal clearance provide significant advantages for biomedicine and pharmaceutical fields.^58–60^

## Conclusions

In summary, we successfully synthesized highly luminescent GSH-protected Au NCs *via* Zn doping. The fluorescence of the fabricated AuZn alloy NCs shows approximately 5-fold enhancement compared with the pure Au NCs. The as-prepared ultrasmall (an average size 1.8 nm) alloy NCs exhibit good photostability and long fluorescence lifetime. Furthermore, experiments based on MTT assays at the cellular level suggest AuZn alloy NCs are of low toxicity and they could significantly enhance the radiation therapy. In addition, *in vivo* experiments indicate AuZn alloy NCs have targeting capability to tumor as well as renal clearance. Nearly 80% of GSH-protected AuZn alloy NCs can be rapidly excreted *via* kidneys in 24 hours, owing to their biocompatible surface ligand and ultrasmall size features.

## Conflicts of interest

There are no conflicts of interest to declare.

## Supplementary Material

RA-008-C7RA13072D-s001
