# Supplementary material for: Fluorescence enhancement of gold nanoclusters via Zn doping for biomedical applications
Source: RSC Adv. 2018 Feb 15;8(14):7396–402. doi: 10.1039/c7ra13072d (PMC9078413; doi:10.1039/c7ra13072d)
Supplement: RA-008-C7RA13072D-s001 [file RA-008-C7RA13072D-s001.pdf]

Electronic Supplementary Material (ESI) for RSC Advances.
This journal is © The Royal Society of Chemistry 2018

Supporting Information

Fluorescence enhancement of gold nanoclusters via Zn doping for biomedical applications

Yanqing Qiao[a], Ying Liu[a], Haixia Liu[a], Yonghui Li[a], Wei Long[b], Junying Wang[a], Xiaoyu Mu[a], Jing Chen[a], Haile Liu[a], Xueting Bai[a], Lingfang Liu[a], Yuan-Ming Sun[b], Qiang Liu[b], Meili Guo[d,*],and Xiao-Dong Zhang[a,c*]

a. Department of Physics and Tianjin Key Laboratory of Low Dimensional Materials Physics and Preparing Technology, School of Sciences, Tianjin University, Tianjin 300350, China.
E-mail: xiaodongzhang@tju.edu.cn;
b. Tianjin Key Laboratory of Molecular Nuclear Medicine, Institute of Radiation Medicine, Chinese Academy of Medical Sciences and Peking Union Medical College, No. 238, Baidi Road, Tianjin 300192, China.
c. Tianjin Collaborative Innovation Center of Chemical Science and Engineering, Tianjin 300072, China.
d. Department of Physics, School of Science, Tianjin Chengjian University, Tianjin 300384, China.
E-mail: meiliguo314@163.com;

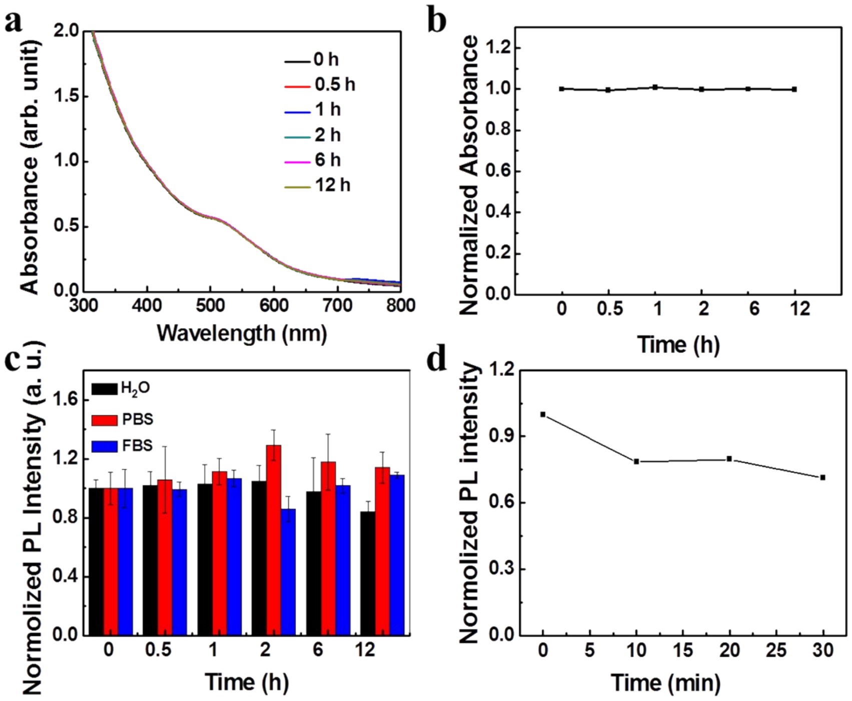

Figure S1 (a) Time-dependent absorption spectrum. (b) Time-dependent normalized absorbance intensity at 525 nm. (c) Photostability of AuZn alloy NCs in H_2O, FBS, and PBS. (d) Photobleaching investigation of AuZn alloy NCs under 30 minutes of continuous laser exposre.

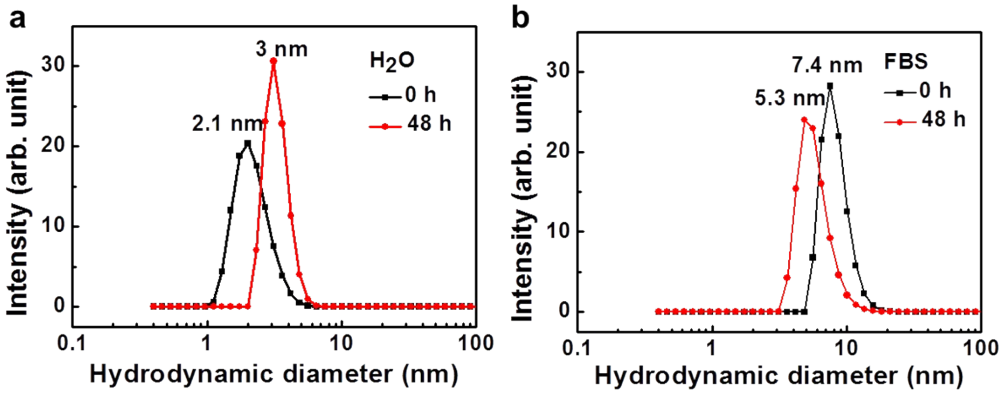

Figure S2 Hydrodynamic size of AuZn alloy NCs in H_2O (a) and FBS (b) measured by DLS at different time point of 0h and 48h.

Table S1 Zeta potential and hydrodynamic size of the Zn-doped Au NCs in H_2O and FBS after 0 and 48 h.

	**0 h**		**48 h**	
	Zeta potential	Hydrodynamic size	Zeta potential	Hydrodynamic size
H_2O	-18.6 mV	2.1 nm	-16.4 mV	3 nm
FBS	-9.4 mV	7.4 nm	-9.44 mV	5.3 nm

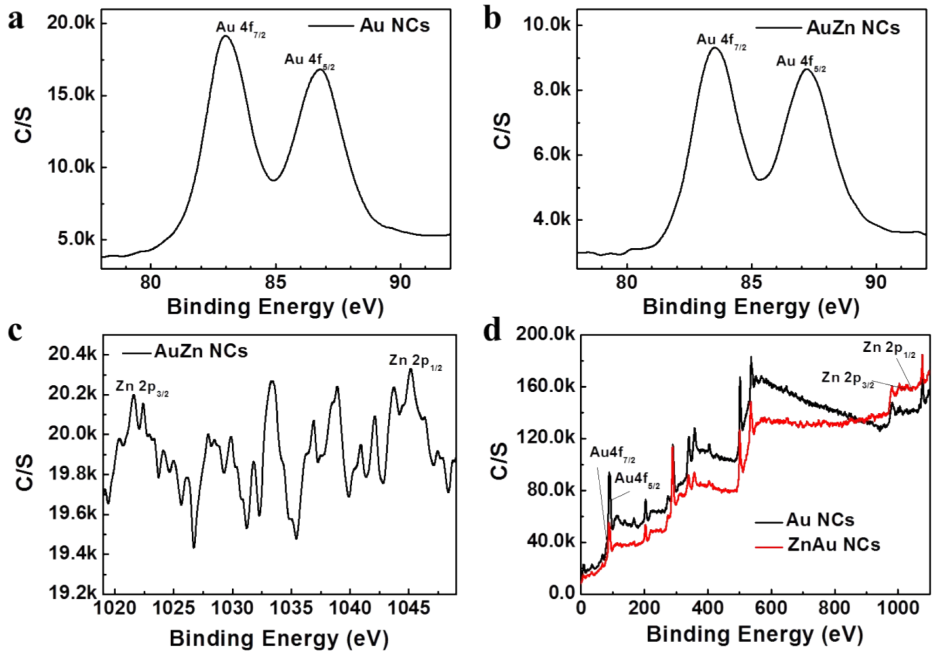

Figure S3. XPS spectra showing the binding energy. Binding energy of Au 4f of (a) Au NCs and (b) AuZn alloy NCs. (c) Binding energy of Zn 2p of AuZn alloy NCs. (d) XPS spectra of Au NCs (rad line) and AuZn alloy NCs (black line).
